# Sociality and temporality in local experiences of distress and healing: Ethnographic research in northern Rwanda

**DOI:** 10.1177/1363461520949670

**Published:** 2020-10-12

**Authors:** Yuko Otake, Teisi Tamming

**Affiliations:** 1 University of Oxford, Oxford, UK; 2 London School of Hygiene & Tropical Medicine, London, UK

**Keywords:** global mental health, healing, hope, local idioms of distress, Rwanda, war and conflict

## Abstract

Prior studies have traced sociality and temporality as significant features of African healing. However, association between the two has not been explicitly investigated. This paper explores how sociality and temporality are associated in local experiences of distress and healing among northern Rwandans. The ethnographic research, including in-depth interviews, focus-group discussions and participant observation, was conducted in 2015–2016, with 43 participants from the Musanze district who have suffered from not only the genocide but also post-genocide massacres. Findings identified common local idioms of distress: *ibikomere* (wounded feelings), *ihungabana* (mental disturbances), *ihahamuka* (trauma), and *kurwara mu mutwe* (illness of the head, severe mental illness). One stage of distress was perceived to develop into another, slightly more serious than the previous. Social isolation played a significant role in the development as it activated ‘remembering’ and ‘thinking too much’ about the past and worsened symptoms. Subsequently, healing was experienced through social reconnection and a shift of time orientation from the past to the future; the healing experience traced a process of leaving the past behind, moving forwards and creating a future through community involvement. The experiences of distress and healing in this population were explained by two axes, i.e. sociality (isolation – reconnection) and temporality (past – future), which are associated with each other. Given the sociality–temporality association in African post-war healing, the study highlights that assistant programmes that facilitate social practice and future creation can be therapeutic and be an alternative for people who cannot benefit from talking-based and trauma-focused approaches.

## Introduction

In the context of post-genocide Rwanda, mental health studies have taken three major approaches: the biomedical, psychosocial, and culturally sensitive (Doná, 2010). Over recent decades, scholars employing these approaches interacted with one another and developed collaborative relationships . The biomedical approach has focused on psychiatric trauma and post-traumatic stress disorder (PTSD) as genocide-caused mental disorders. Taking a positivist view that sees a disease as an objective reality rather than a social construct, an epidemiological study reported probable PTSD in 62% of the population two years after the genocide (Neugebauer et al., 2009). Subsequently, psychological programmes targeting trauma and PTSD were initiated (Chauvin, Mugaju & Comlavi, 1998; Neuner et al., 2004, 2008; Schaal, Elbert & Neuner, 2009). Whereas some studies reported positive impacts of psychological programmes (Neuner et al., 2004; Neuner et al., 2008, Shaal et al., 2009), others noted negative consequences and critically reflected cultural insensitivity of these programmes (Kumar et al., 1996; Neugebauer, 2006). One critical appraisal of biomedically oriented programmes pointed out that they focused on individual psychology, and had thus insufficiently considered social aspects of mental health (Bracken, Giller & Summerfield, 1995). Other critiques have argued that the Western psychiatric concepts of trauma and PTSD do not perfectly fit local populations, and that distress expressed through local concepts of mental health embedded in culture and context may have been missed (Kumar et al., 1996, Neugebauer 2006).

To surmount the these issues, psychosocial and culturally-sensitive approaches have been developed. Psychosocial approaches have emphasised social aspects of trauma such as isolation, discrimination and financial burden (Rutayisire & Richters, 2014), along with the implementation of community-based and resilience-oriented programmes to facilitate social support and healing (King, 2014; Scholte et al., 2011). Such approaches have reported therapeutic effectiveness for treatment of common mental disorders, as well as improved dignity, trust and reconciliation (Richters et al., 2008, 2010, Scholte et al., 2011). Meanwhile, culturally-sensitive approaches have documented local idioms of distress, drawing from anthropological perspectives. The most well-known is ‘*ihahamuka*’, the experience of shortness of breath and frequent fear, which was suggested as corresponding to the Western psychiatric construct of ‘trauma’ (Hagengimana & Hinton, 2009). Since *ihahamuka* is widely reported among genocide survivors, many researchers have discussed the concept as a representation of genocide trauma. Other reported idioms include *agahinda* (sadness/sorrow/grief), *akababaro* (depression), *kwiheba* (hopelessness), and *guhangayika* (stress/worry) (Bolton, 2001, Betancourt et al., 2011). The authors who documented these idioms suggest that they are similar to Western psychiatric concepts of depression and anxiety disorders including PTSD, but also contain important culture-specific features. These studies suggest that understanding locally-conceptualised idioms is important when planning and conducting intervention programmes (Betancourt et al., 2011; Bolton et al., 2007).

The studies reviewed above have advanced the understanding of locally-conceptualised mental health problems embedded in Rwandan culture and context. However, the majority of studies focus on genocide trauma. As such, the suffering of other populations marginalised by the mainstream genocide trauma narrative remains under-studied. Rwandan post-genocide policy has constructed the genocide trauma narrative as a symbol of national suffering (Doná 2019). Accumulated research and knowledge on genocide trauma has contributed to this narrative construction. Consequently, the concept of trauma, or *ihahamuka*, became strongly associated with the genocide, marginalising and silencing the suffering of other populations (Longman & Rutagengwa 2004). Locally-experienced atrocities and forms of distress are in fact wide-ranging; for example, discrimination against, and stigmatisation of ex-prisoners (Rutayisire & Richters 2014), silenced suffering from refugee experiences in eastern Zaire (now eastern DRC) and post-genocide massacres in Northwestern Rwanda (Burnet, 2012; Otake, 2019), and HIV/AIDS-related distress (Betancourt et al., 2011). Given these limitations to existing research in the post-war context, it is important to illuminate marginalised experiences and provide a broader, more socioculturally-sensitive understanding of mental health among Rwandans.

### Local experiences of healing in Sub-Saharan Africa, sociality and temporality

While a body of literature has investigated distress among Rwandans, little research has examined healing. Sociality and temporality in particular have yet to be investigated as common constituents of healing in this context. We define ‘sociality’ and ‘temporality’ as follows, drawing from phenomenological thinking (Schütz, 1962; Ricœur, 1991) and anthropology of African healing (Feierman & Janzen, 1992; Last, 2000; Jackson, 2004); ‘sociality’ refers to the experience of social connectedness through interacting, living or being with others. ‘Temporality’ refers to the orientation of experience to a temporal frame, through remembering the past, experiencing the present, or imagining the future.

Although we should acknowledge the reality that some sufferers have had insufficient opportunities to join in social group or assistance programmes, community and social groups have played an important role in Rwandans’ healing. One typical healing process related to sociality is sharing verbal and non-verbal narratives, e.g. sharing stories, dance and music, gifts, and everyday life, resulting in nurturing, mutual understanding, trust and solidarity (King, 2014; Burnet, 2012; Leonard, McKnight, Spyrou & Pells, 2011; Otake, 2018). Meanwhile, a few studies also describe healing processes related to temporality; for example, suffers begin to take a distance from the traumatic past and create a hopeful future (Dushimirimana, Sezibera & Auerbach, 2014, Leonard et al., 2011).

In the wider setting of Africa, a classic ethnography by Feierman & Janzen (1992) demonstrated that illness and healing are constructed in social context, providing a view of health as a process maintained by social support within kin and community networks .Other anthropological literature has noted the role of multiple temporalities (de Jong, 1987; Igreja, 2003, 2012). For example, Igreja (2012) and Igreja & Dias-Lambranca (2006) showed that Mozambicans live in multiple temporalities, and draw on diverse important sources of time orientation including nature, spirits and dreams, which diverge from the linear and mechanical sense of time found in many Western societies. In the Mozambican cultural context, healing was also described as showing multiple temporalities with healers and sufferers moving backwards and forward in time whilst experiencing imaginary dimensions of time. In these experiences, pre-war, war and post-war times were linking with the healing of sufferers (Igreja, 2003, 2012).

### Objectives

Prior studies have traced sociality and temporality as significant features of African healing. However, association between the two has not been explicitly investigated. This study explores: (1) local experiences of distress and healing in a population marginalised by the mainstream genocide trauma narrative; and (2) how sociality and temporality are associated in local experiences of distress and healing. The study aims to contribute to advancing the understanding of locally-experienced distress and healing, and provide information for the development of future assistance programmes.

## Methods

### Research site

Rwanda experienced a series of civil wars and genocide in the 1990s. The conflicts were deeply rooted in regimes of German and Belgian colonisation that had allocated ruling positions to Tutsis under the Tutsi monarchy (Prunier, 1995). The suppressed Hutus revolted and created an independent, Hutu-dominated republic (1959–1961), during which Tutsi elites were expelled from the country. The second generation of exiled Tutsis formed the Rwandan Patriotic Front (RPF) and began a civil war to reclaim the country (1990–1994). At the end of this war, the Hutu-led government and militias waged genocide against Tutsis and massacred moderate Hutus (1994) (Des Forges, 1999; Prunier, 1995). The victorious RPF eventually ended the war and genocide and Tutsis regained control of Rwanda.

People in Rwanda (including Hutus who were involved in the genocide, Hutus who were *not* involved in the genocide, and Tutsis) fled to eastern Zaire (now eastern DRC) to avoid political chaos and formed refugee camps. The RPF army perpetrated further massacres against the Hutu refugees and forcibly closed the camps (1994–1997) (Human Rights Watch, 1999; UNHCHR, 2010). Unarmed civilian Hutus fled back to Rwanda, whilst other Hutus rearmed in eastern Zaire and began an insurgency in northwest Rwanda. The RPF called the latter ‘*abacengezi*^1^ (infiltrators)’ and deployed counter-insurgency operations; this developed into the *abacengezi* war, or ‘the insurgency in the northwest’ (1997–2000) (Amnesty International, 1997, 1998 *;* African Rights, 1999, Human Rights Watch, 1999). During this war, civilians in northwest Rwanda were massacred by both RPF and *abacengezi* forces for being suspected of supporting the opposition (Amnesty International, 1997, 1998; Human Rights Watch, 1999; Reyntjens, 2013). The northwest was closed as a ‘hazardous area’ and international aid could not reach there until 2003. (See *Otake* 2019 for a review of further post-genocide events.)

The research site, the Musanze district, is part of northwest Rwanda. It has had an extremely high proportion of Hutus since before colonisation (Newbury,1980). According to the 1991 pre-genocide census, only 0.5% of Musanze citizens were Tutsi, much lower than the 10–15% in the general population (Fujii, 2009). Given the extremely small proportion of Tutsi, the genocide was much less active in Musanze than other parts of the country. The number of genocide survivors in Musanze was in fact low: 1,893, only 0.6% of all genocide survivors across the country (INSR, 2008). However, Musanze citizens were summarily slaughtered during other events, including the forced displacement in eastern Zaire and the *abacengezi* war (Amnesty International, 1997, 1998; African Rights, 1999; Human Rights Watch, 1999). No ofﬁcial data on the victims exist, but the district survey traced its impact eight years after the end of the war. 21% of children in Musanze were orphans (NISR, 2008), which was 5% higher than the national average (NISR,2010/2011).

In short, the majority of Musanze citizens were victimised by the post-genocide massacres, rather than the genocide in 1994. Hence, this victim population in the Musanze district is marginalised by the national narrative of genocide trauma, as well as by genocide trauma research; information on their mental distress and healing is scarce.

### Research team

The first author conducted ethnographic research in Musanze (August 2015–May 2016), built on her prior life and work experience in the field (July 2010–August 2012). Seven Musanze residents received research training and worked as research assistants. Amongst them, a Tutsi man had full involvement, being present at most interviews as an interpreter and checking the quality of all transcriptions and translations with the first author. As a local non-governmental organization officer, he had wide-ranging networks and friendship with other residents regardless of ethnicity^2^, gender and socio-economic status; participants trusted him and talked to him openly and comfortably. Additionally, the first author’s background as Japanese woman who is neither politically nor historically related to any ethnic identities or political parties, enabled building trust with assistants and participants, and facilitated their rich narratives.

### Participants and sampling

Participants were approached through the first author and assistants’ networks in villages. Since local post-war context is still fragile in Rwanda, trust built on existing networks was vital to gain access to participants’ narratives. Any Musanze citizens aged 20 or over were included as participants; those who were based in institutions (e.g., prison, political parties) were excluded. The initial sampling began in the area showing the highest percentage of orphans in the recent district survey (NISR, 2012) as a benchmark for massacres. The next sampling phase extended to neighbouring areas to include a maximum variation sample of participants' characteristics (i.e., age, gender, ethnicity, occupation, socio-economic status, home village). After the initial analysis, the research moved to theoretical sampling which sought relevant data to examine coding schemes, analytical questions and provisional hypotheses which had emerged from the initial analysis. The sampling was terminated when new data began to exceed the research scope, not just repeat the obtained data, which indicates ‘saturation’ (Charmaz, 2006).

A total of 43 participants joined in-depth interviews and focus-group discussions (FGDs) but three did not complete interviews; hence, data from 40 participants (aged from 22 to 84 years) were analysed (Table 1). Among them, 60% were women, 80% were Hutus before 1994, 35% were subsistence farmers and 57.5% had stable cash income through employment or their own small-businesses. Some were repeatedly interviewed to collect further information. Data for analysis included 70 interviews, three FGDs, and fieldnotes generated from participant observation of social groups and everyday life in the village. Respecting the local culture of gift exchange, all participants were given two kilograms of rice (approximately $1.5, August 2015) as an honorarium for participation.

**Table 1.** Characteristics of research participants (*N* = 40).

	***n***	**%**
Gender		
Female	24	60
Male	16	40
Age (range from 22 to 84 years)		
20–29 years	8	20
30–39 years	17	42.5
40 years and over	15	37.5
Ethnic identity before 1994*		
Tutsi	8	20
Hutu	32	80
Occupation		
Subsistence farmer	14	35
Small business owner	5	12.5
Paid worker**	4	10
Non-governmental organisation officer	4	10
Landlord/landlady	3	7.5
Security guard	3	7.5
Student	3	7.5
Government officer	2	5
School teacher	2	5

*Information about pre-genocide ethnicity was provided by local assistants who live in the same village as participants, given that the law of Rwanda prohibits asking about an individual’s ethnicity; the research involved a higher proportion of Tutsi (20%) than in the Musanze population (0.5%, based on the pre-genocide census in Fujii 2009).

**Cook, bike rider, tailor, and mason.

### Data generation and analysis

Except from four interviews (in English), all the interviews and FGDs were conducted in Kinyarwanda. Although the first author speaks Kinyarwanda, an assistant acted as an interview interpreter since there had been suspicions that the first author might be a government inspector or collaborating with extremists when she had conducted Kinyarwanda interviews by herself. Topic guides were produced through close discussions with assistants and then pre-tested with a few participants to ensure that wordings are comprehensible for them. The topic guides were designed to be loosely structured and conversational, using three key questions to ask about distress and healing (Table 2). All interviews and FGDs were transcribed and double checked by the first author and assistants according to agreed guidelines. All data (transcriptions, fieldnotes) were manually analysed; coding schemes were developed and reﬁned through the cyclical process of theoretical sampling, analyses, constant comparison, and memo-writing. Common concepts of distress and healing as well as common social groups for healing emerged from participants’ narratives; then theoretical sampling sought further narratives to explore these common themes. Finally, ‘member check’ (Charmaz, 2006) with assistants and participants ensured representation of their subjectivity in the analysis. All data generation and analysis followed grounded-theory methods (Glaser & Strauss 1967, Charmaz, 2006).

**Table 2.** Interview topic guide.

Key questions
1) Can you tell me your experience during wartime and how you have survived until today?
2) Can you tell me your experience of how other people helped you with the reconstruction of your life or recovery of your heart?
3) Can you tell me your experience of how your community/group helped you with the reconstruction of your life or recovery of your heart?

### Ethics

The study was approved by the Rwanda National Ethics Committee (No. 339), the Ministry of Education (No. 2944), and the Ethics Committee of London School of Hygiene & Tropical Medicine (LSHTM Ethics Reef: 9182), as part of larger ethnographic research on community resilience in northern Rwanda (Otake, 2017). All quotations in this paper use pseudonyms.

## Findings

### Local experiences of distress: Social isolation and past-oriented distress

Participants reported having experienced four tragedies during 1990s: the civil war (1990–1994), the Tutsi genocide (1994), forced displacement in eastern Zaire (1994–1997), and the *abacengezi* war (1997–2000). Given the extremely small proportion of Tutsis (0.5% in 1991, Fujii, 2009), the genocide was reported to be much less active in Musanze than other parts of the country. While several participants reported witnessing the genocide near the central town and in Kigali, some Tutsis reported that no-one was killed during the genocide in their village. Meanwhile, the *abacengezi* war (1997–2000) was commonly reported to be the most calamitous incident since Musanze was part of the main battleﬁeld and massacres against civilians, regardless of ethnicity, took place on daily basis.

Except three who reported no family loss, all participants reported having lost around five (the least being one, the most 18) family members (*umuryango*, referring to kin members who live in the same kin compound), while having risked their own lives and narrowly survived massacres during the *abacengezi* war. At least three women reported having been raped or sexually assaulted as teenage girls or even younger during the same war. All who had lost family members perceived that loss of family was the main cause of distress. By contrast, the three exceptions reported no distress, which was attributed to no family loss.

We identified four common concepts of distress in participants’ narratives: *ibikomere* (wounded feelings), *ihungabana* (mental disturbances), *ihahamuka* (trauma), and *kurwara mu mutwe* (illness of the head) (Table 3). While most participants reported experiences of *ibikomere* (*n* = 37), only a few reported *ihungabana* (*n* = 4), *ihahamuka* (*n* = 1), and *kurwara mu mutwe* (*n* = 2), as their own experience. One possible reason for the few reports was that the latter three refer to behavioural manifestations which can be stigmatised, or that the prevalence was in fact low as they were mild to severe mental illnesses. To supplement the data, two focus-group discussions with neighbourhood groups were formed and their views on these three conditions were asked for. We will illustrate each concept and how one concept develops into another, drawing from participants’ narratives.

**Table 3.** Local idioms of distress in participants’ narratives

**Idioms of distress and reported symptoms**
*Ibikomere(n.)* (wounded feelings)*
*Wenyine(pro.)* (feeling alone)
*Kubabara(v.)* (feeling sad, pain)
*Intimba(n.)* (deep sorrow)
*Agahinda(n.)* (depression)
*Kwiheba(n.)* (hopelessness, despair)
*Guhangayika(v.)* (being anxious, worried)
*Ubwoba(n.)* (fear)
*Kwishisha(n.)* (mistrust)
*Ihungabana(n.)* (mental disturbances)**
*Kwigunga* (social withdrawal)
*Ihahamuka(n.)* (trauma)**
*Kwigunga* (social withdrawal)
*Kujunjama* (mutism)
*Kurwara mu mutwe(v.)* (illness of the head)***
*Kwigunga* (social withdrawal)
*Kujunjama* (mutism)
*Kwiruka* (running, agitation)
*Kurota nabi* (nightmare)
*Kurotaguzwa* (hallucination)

Following embodiments were also reported or observed**:**

*Embodied feelings; e.g. sad face, head in hands, eyes filled with tears, strongly-stressed words due to anger.

**Deviant behaviour; e.g. inappropriate responses in communication, continuous crying, violent behaviour.

***Deviant behaviour; e.g. violent behaviour.

### Ibikomere

*Ibikomere* (wounded feelings) literally means physical or mental ‘wounds’; when it is used for mental wounds, it refers to negative feelings resulting from tragic events. *Ibikomere* is a countable noun; the singular form is ‘*igikomere*’. Tragic events and *ibikomere* were inseparable in participants’ narratives and they counted a combination of tragic event and resulted in *ibikomere*, shifting from one combination to another. *Ibikomere* was commonly explained as “something in the heart”; a man compared it to bodily injury when someone is shot, but “it is a feeling, something inside” (30s, fieldnotes, 25-Apr-2016).

Reported *ibikomere* feelings included *kubabara* (sadness), *intimba* (deep sorrow), *agahinda* (depression), *kwiheba* (hopelessness/despair), *guhangayika* (anxiety/worry), *ubwoba* (fear), and *kwishisha* (mistrust). Depressive feelings were sometimes unspoken but embodied in a sad face, a head in the hands, or eyes filled with tears. Anger was also observed to be suppressed and expressed implicitly, for example stressing words particularly strongly while talking about perpetrators, or saying “I can’t forgive any of them” (woman, 20s, interview, 26-Mar-2016). Meanwhile, some narratives of *ibikomere* were emotionless, focusing only on events.

The most commonly-reported *ibikomere* were feelings of social isolation and grief, i.e., isolation, loneliness, and helplessness, due to the loss of family members. One woman spoke about her experience of surviving massacres during the *abacengezi* war, which resulted in her feeling of isolation and deep grief:

*Igikomere* that I will *never forget* is… Can you imagine that you had lived with many neighbours and you see all of them were killed and stay alone in that area? I can *never forget* this situation in my life. […] Many siblings and friends died and I stay… I stay with few of them. I have only few of them survived. (40s, interview, 16-December-2015)

As she narrated to ‘*never forget*’, *ibikomere* was always reported to arise from ‘remembering’ the past. When sufferers are socially isolated and remembering the past, *ibikomere* often progressed to the more severe mental health conditions as follows.

### Ihungabana and ihahamuka

*Ihungabana* (mental disturbances) refers to behavioural problems resulting from *ibikomere*. Reported symptoms of *ihungabana* include social withdrawal, crying continuously, violent behaviour, and inappropriate responses in conversation. From the participants’ point of view, the distinct difference between *ibikomere* and *ihungabana* was that the former are invisible emotional problems whereas the latter are visible behavioural problems:

[If you have *ihungabana*, someone else] can see that you have something which is not good. You may beat other people, cry, or speak with wrong voice. […] You can see the sign. But for someone who has [only] *ibikomere*, it’s just inside. […] You can’t see the sign. (man, 30s, interview, 10-May-2016)

Some participants and assistants perceived *ihungabana* as identical to ‘trauma’. However, in post-genocide Rwanda, the formal translation of ‘trauma’ is ‘*ihahamuka*’.

*Ihahamuka* (trauma) refers to feeling ‘breathless with frequent fear’ (Wulsin & Hagengimana, 1998), originated from Bantu root words that indicate the absence of inhalation or a state of not breathing (Wilson & Lindy, 2013). The word is officially and academically acknowledged as the local translation of the psychiatric ‘trauma’. Particularly, it is well-known as a local representation of genocide-caused trauma, including physiological and psychological symptoms (Hagengimana & Hinton, 2009).

However, in this research, the word *ihahamuka* very rarely emerged. The majority of participants were unfamiliar with this word. Even if they had heard it, they thought it was for genocide survivors, not for themselves. Only a few participants, who had been trained on genocide trauma at school or by international aid organisations, talked about *ihahamuka* in interviews. Some of them saw *ihahamuka* as an exacerbated condition of *ihungabana*, while others saw *ihahamuka*as as synonymous with *ihungabana*. Trauma psychoeducation programmes’ facilitators in Musanze as well as in Kigali told that people in the village are familiar with *ihungabana* and unfamiliar with *ihahamuka*, therefore, they need to teach the concept of trauma, *ihahamuka*, through psychoeducation programmes.

Overall, both *ihungabana* and *ihahamuka* were perceived as behavioural manifestations of *ibikomere*. Their symptoms were mutually characterised by social maladaptation, impaired communication, and deviant behaviours. However, *ihahamuka* was explained as a worsened condition of *ihungabana* with more explicit symptoms. Participants believed that *ihungabana* can be healed by family and community care, whereas *ihahamuka* needs bio-psychological treatments, including psychological first aid for trauma and psychiatric drugs. Importantly, at the stages of *ihungabana* and *ihahamuka*, sufferers do not only ‘remember’, but also ‘think too much’ about their lost family members and their own life. The experience of *ibikomere* was described as growing when sufferers remember, and further, think too much about the past when they are isolated from society. This development then leads to the following final condition.

### Kurwara mu mutwe

*Kurwara mu mutwe* (illness of the head) is the final stage of the development of *ibikomere*, which is perceived as a severe mental illness resulting from aggravated *ihahamuka*. Reported symptoms included *kurota nabi* (nightmare) and *kurotaguzwa* (hallucination) (both are idioms comprising the word ‘*kurota* [having dreams]’), *kwigunga* (withdrawal), *kujunjama* (mutism), and ‘*kwiruka* (running)’. *Kwiruka* (running) is a metaphor for nonsensical behaviour due to agitation, e.g., suddenly leaving the house and running into the road or around the village for no reason. People who have *kurwara mu mutwe* also showed extreme tension and nervousness. Sometimes they were called *umusazi* (a mad person) among community members. Although no clear perceived discrimination between them, those who have a reason for showing symptoms (e.g., a history of victimisation) were seen to have ‘illness of the head’ rather than being ‘mad’.

Participants perceived *kurwara mu mutwe*as requiring specific treatments with traditional medicine, including herbal medicine, counselling and family consultation, or Western medicine, including medical tests (e.g., brain scan) and pharmaceutical treatment. Most commonly, family and community members take the patient directly to a traditional healer; some Christians choose Western medicine at health centre and hospital following the church’s health education. At the hospital, a medical doctor prescribes psychotropic drugs if a psychiatric problem is diagnosed; however, the medical doctor sends the patient to a traditional healer if spirit possession caused by Satan or bad spirits is suspected.

Like other concepts, *kurwara mu mutwe* also showed orientation towards the past. A man who previously suffered from *kurwara mu mutwe* expressed his experience as the ‘illness of thinking a lot’ about the past:

[There are] things that take me like… having nightmares (*kurota nabi*)… taking me far away in things… people who died long ago… they died… how things did. Things [the illness] arrived [to me] after that. Just like there is an illness of thinking that I don’t know…[…] especially having hallucinations (*kurotaguzwa*), dreaming about cemeteries, things like that. I don’t know. Just it’s so many… (30s, interview, 28-Oct-2015)

During this man’s illness, unknown entities were coming and taking him far away to a place in the past, which was associated with the land of the dead. The past memories intruded uncontrollably in his thoughts; the symptom of ‘thinking too much’ about the past was the severest in all the distress conditions. Besides, as indicated by his repeated words ‘I don’t know how to explain’ with many pauses and interruptions in his speech, his suffering, which was experienced in a different space and time from the here and now, was too profound to be verbalised and shared with others. Due to the difficulty in sharing subjective experience with others, people who have *kurwara mu mutwe* are socially withdrawn and haunted by the past.

### Development of distress

**Figure 1.**
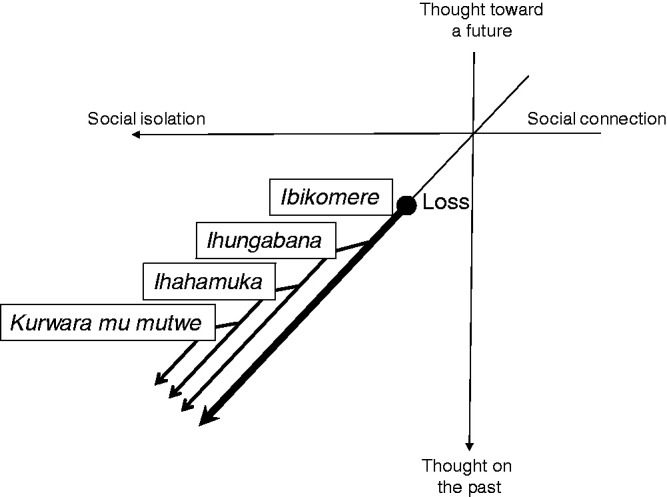
Local experiences of distress and their development in sociality and temporality.

Participants explained that *ibikomere* develops into *ihungabana*, then *ihahamuka*, and finally into *kurwara mu mutwe* (Figure 1). In their views, one concept develops into the next which is slightly more serious than the last, while the *ibikomere* feelings are sustained throughout this development. The latter three conditions are behavioural manifestations of *ibikomere*; without *ibikomere*, the others cannot develop. In other words, *ibikomere* is a prerequisite for the development of others, thus for the participants, it was the most important.

The development of *ibikomere* was explained as follows from their point of view: *ibikomere* is first caused by a loss, mostly collective loss. The loss itself also puts the person into socially-isolated circumstances; at the same time, the person becomes prone to be withdrawn and isolated due to his/her *ibikomere*. These social isolations, led by the loss and withdrawal, trigger remembrance and thoughts of the loss, and consequently, worsens *ibikomere* and develops severer conditions.

For the participants, social isolation and past-oriented thoughts were associated with each other; the more participants experience isolation from society, the more they are haunted by memories and thoughts of the past. Participants described *ibikomere* as arising when they remember and think too much about their lost family members; e.g., “if someone remembers it, *ibikomere* also comes” (man, 50s, interview 9-April-2016) and “I always think about them [lost family members] and it makes my *ibikomere*” (woman, 70s, interview 31-March-2016). Such remembering and excessive thinking were initiated and also reinforced when people are isolated from society. One young woman explained how *ibikomere* develops into *ihungabana* and *ihahamuka*:

If you stay alone here [at home], you cannot help but remember it. […] When I am with many people or when I am talking to some people, I don’t remember a lot. But when I am alone, I think about my life [and *ibikomere* becomes more severe]. (20s, interview, 29-November-2015).

Similarly, *kurwara mu mutwe* was also explained as developing through social isolation and excessive thinking. Another woman explained her neighbour’s *kurwara mu mutwe* symptoms: “being withdrawn and living alone, they are all combined. Maybe this [combination] is the cause of her *kwiruka* [running; a symptom of *kurwara mu mutwe*]” (40s, FGD, 21 December 2015). Another man in the same focus group added: “When you are withdrawn, the brain starts thinking too much and cycling too much. After that the brain becomes to be like broken” (30s, FGD, 21-December-2015).

In short, local experiences of distress have two characteristics: social isolation and past-oriented thoughts. In the *ibikomere* stage, sufferers have a feeling of isolation due to family loss but are not necessarily disconnected from society. They ‘remember’ the past on occasions when they are isolated. Once it develops into *ihungabana* and *ihahamuka*, sufferers are socially withdrawn and ‘think too much’ about the past. In the final stage of *kurwara mu mutwe*, sufferers are not only withdrawn, but also unable to communicate with others; almost disconnected from society, they are haunted by the past. In this development process, social isolation is likely to be a key to initiating and also reinforcing ‘remembering’ and ‘thinking too much’ about the past, while facilitating a more rigid orientation towards the past.

### Local experiences of healing: Social reconnection and future-oriented healing

Whereas social isolation plays a key role in developing the locally-perceived distress after war, social reconnection opens a window to the healing process, through which sufferers shift their orientation from the past to the future. Healing typically takes place through participation in a community or social group (we use these terms interchangeably). We identified faith-based groups and mutual-saving groups as the most common social groups that have supported the recovery, reported by 17 and 11 participants respectively; six reported both groups. Although only three mentioned kinship and neighbourhood groups, they were a foundation of all the other groups within a geographical area. Other social groups were also reported but few: government-led associations (*n* = 3), intervention programmes provided by international organisations (*n* = 2), and genocide survivor association (*n* = 1).

Participants reported wide-ranging local concepts that can represent ‘healing’ (Table 4).In the early stage of the research, they reported concepts that are used by the government, such as *isanamitima* (recovery of the heart, mental recovery) and *ukwiyubaka* (reconstruct oneself, resilience). *Isanamitima* is an idiom composed of *gusana* (mending) and *imitima* (hearts). Meanwhile, *ukwiyubaka* is the word improvised by the government to represent the foreign concept of ‘resilience’ and officially used to signify the resilience of genocide survivors (*The New Times*, 2014). The origin of the word is *kubaka* (building, constructing), which can widely encompass social, economic, physical and mental reconstruction. As the research continued, however, more diverse expressions appeared in interviews and conversations. The most recurrent ones formed idioms with *ibikomere*; e.g. *kugabanuka ibikomere* (reducing wounded feelings) and *gukira ibikomere* (healing wounded feelings). Using these idioms, participants explained that ‘trying to forget’ or ‘stop thinking about the past’ are key to healing. By contrast, when thoughts or memories of the past occasionally returned and disturbed them, they perceived their healing process to be still ongoing.

**Table 4.** Local idioms of healing in participants’ narratives*

Idioms with *ibikomere*(wounded feelings)
*Gukira ibikomere* (healing wounded feelings)
*Kugabanuka kw’ibikomere* (reducing wounded feelings)
*Ibikomere bigenda bisibangana* (wounded feelings are reducing)
*Gusibangana ibikomere* (cleaning wounded feelings)
*Kurangira kw’ibikomere* (endingwounded feelings)
*Kugenda kw'ibikomere* (wounded feelings are going away)
*Kurenga bya ibikomere* (going beyondwounded feelings)
Idioms with *umutima(sn.)/imitima(pl.)* (heart, hearts)
*Isanamitima* (recovery of the heart, mental recovery)
*Gusana imitima* (rebuilding or repairing the hearts)
*Gusubira mu gitereko* (the heart gets back into the right place)**
Other important idioms
*Ukwiyubaka* (reconstruct oneself, resilience)
*Kwiyunga* (repairing relationship, reconciliation)
*Kuva muri ibyo bibazo* (leaving those problems)

*Using these idioms, participants explained healing as leaving the past and moving towards a future.

**In the Rwandan view, when someone is hurt, his/her heart beats (palpitations), trying to move out of the body; when s/he is healed, the heart moves back into the right place and one no longer feels the beats.

It is noteworthy that participants’ perceptions of healing may be different from the official government narrative. The Rwandan government propounds ‘*Kwibuka* – Remember the Genocide against the Tutsi’ as one central policy to reconstruct the country, where remembrance is particularly emphasised to prevent the repetition of genocide. However, in this research, only a few participants, who had received training on the government’s political ideology in *ingando*re-education camps as public workers or ex-prisoners, narrated the importance of remembrance. Even these participants remarked on the significance of ‘trying to forget’ at the same time; “We have to remember but also we should not remain the slaves of history. […] We have to try to forget […], try to put them aside, and decide to do something else in order to develop ourselves.” (man, 20s, interview 19-Nov-2015).

Importantly, ‘trying to forget’ or ‘stop thinking about the past’ were very frequently related to ‘thinking about the future’ through participating in communal activities;

You can’t remove it [the past] out of yourself. But don’t think about it, and it brings peace in you.[…] Some people have hope through communal prayer, others through having a new friend. If you lost your family, you create another family as you want. For me, [mental recovery is] not to think about the past, not to remember the past, but to make a decision to see a future. (woman, 20s, interview 20-Apr-2016)

Meanwhile, another woman recounted the importance of ‘being with’ sufferers to support them. Explaining how she comforts her husband and his family members, she said:

When someone starts thinking about this kind of situation [the loss of family members], […] you should comfort people and be with them. [… Then] they can reconstruct themselves (*ukwiyubaka*) and think about a future. (30s, interview, 20-Dec-2015)

**Figure 2.**
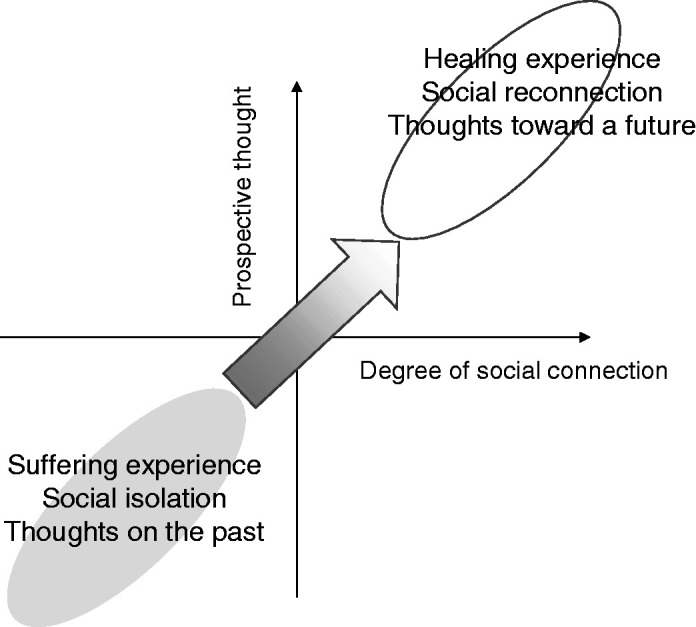
Local experiences of healing to cope with the distress states in sociality and temporality.

As shown above, participants commonly described the healing pathways as a time trajectory of leaving the past and moving toward a future through living with others (Figure 2). In the following sections, we will illustrate this time trajectory using case stories of Namahoro and Didiher, as the most typical and representative narratives of healing pathways with the most commonly-reported therapeutic social groups: faith-based and mutual-saving groups. 

### Narrative of healing and time trajectory I: A faith-based group

Namahoro is a widow in her early 40s. She lost her husband during the *abacengezi* war when she was in her 20s; later she became a member of a faith-based group for widows in Musanze, which helped her to heal the wounds of war. She described how she had suffered before joining in the group:

I was thinking in my heart that I stay alone [at home] with my children. […] I had no hope in my heart and I was overwhelmed alone with problems [of raising three small children by myself]. (interview, 28-Oct-2015)

She experienced a typical condition of *ibikomere*: loneliness, helplessness, social isolation and withdrawal. At this stage, ‘children’ were recounted as a symbol of ‘problems’ brought by the loss of her husband in the past.

However, one day she happened to see members of a widows’ group making pledges in a mass, which strongly appealed to her. She began to attend the group’s masses and gradually found: “the lessons [in the mass] can comfort me and reconstruct our hearts.” Finally, she bought the group book, which contained ‘lessons’ and members’ narratives of recovery, and then became a formal member. She talked about the gradual shift in her focus from the past to the future through community involvement:

I left home to go to the church with a sad heart. When I arrived there, […] a facilitator gave some examples of a person [a Saint] who had problems bigger than mine. […] I also saw other members of the community who had problems bigger than mine. I saw someone who was left alone without her child. [… But] I still have my children. God continues to protect my children and I will see them growing up. I thanked God and I went home with happiness in my heart. […] The group helped me to recover very much. (interview, 28-Oct-2015)

Like many other participants, Namahoro recounted her awareness that everyone has at least one problem which is worse than those of others, and said: ‘I’m not the only one who suffers.’ The faith-based widows’ group provided Namahoro with an opportunity for social reconnection, through communal prayer and talking to others about Christianity. The group also provided master narratives of recovery through ‘lessons’ in the mass and in the book, which guided her healing processes. Consequently, the meaning of ‘children’ shifted from ‘problems’ brought by the past to ‘growing up’ toward the future.

Additionally, the more she was involved in the group, the more time she spent on social activities organised by the group, such as cleaning church buildings and visiting vulnerable people:

I used to go to church only on Sundays. But after I joined in the group, I began to go to church on Wednesdays [too, to participate in the community activities]. […] When there are some activities, I went there three times in a week. All of those activities made me recovered. They help me not to stay [withdrawn at home] for personal activities. (interview, 28-Oct-2015)

Moreover, the increased social time changed her thoughts to be future-oriented in order to remember the next meeting agenda:

Once you start participating in a community, then you start a countdown of days until the next meeting. […] Today, I went to pray at the church, then after three days I get a meeting of this group, and two days later I get a meeting of that group, like that. Think about the next meetings all the time, and you get a calendar in your heart. (fieldnotes, 21-Dec-2015)

In this way, her thoughts that had previously dwelt on the past were transferred to the future. Namahoro’s story exemplifies one way in which people who are haunted by the past can move forward through community involvement.

### Narrative of healing and time trajectory II: A mutual-saving group

While faith-based groups generate a time trajectory through social activities, mutual-saving groups do so through money and collective saving. One exemplar narrative was provided by Didier, a male banana beer trader in his late 20s. His elder brother had been killed during the *abacengezi* war; after that he inherited his bar and then joined a mutual-saving group. He recounted how the group has healed his mental wounds, particularly through *ubusabane*, a convivial party following monetary transactions in regular meetings:

The group plays a role in mental recovery because when you meet a lot of people, you talk to them [about your problems] and they give you advice on the life of tomorrow. […] That’s to say, the group is not only for money […] but it also helps us to meet people; we talk to each other about the everyday life. It is very, very important. (interview, 17-Nov-2015)

*Ubusabane* was commonly perceived as the most important part of the meeting, during which members drink together, traditionally local homemade banana beer, and chat with each other. This is the time they share problems of life and business, and discuss how to resolve them through communal effort; if needed, they take collective action to tackle a member’s problem. With highly organised systems of monetary transactions and *ubusabane*, mutual-saving groups reconnect isolated people and produce a tangible future with livelihood development.

While *ubusabane* and mutual help healed Didier’s wounds, the mutual saving enabled him to make a future plan for his life and put it into practice:

When a turn to take money comes, they [leaders] give you around 300,000 FRW [approximately $390, August 2015]. […] I use it for my future life. I can buy a farm […] or roofs so that I will be able to build a house when the future comes. This is another reason why I like this group. They give you enough money to do something. It helps you to do a visible [tangible] activity. (interview, 17-Nov-2015)

Through participation in a mutual-saving group, members gradually become able to make future plans, start counting the days to the next allocation of money, and realise their plans in a practical and tangible way. The credibility of the mutual-saving system maybe an important constituent of this healing pathway; for example, the method of collecting and distributing money is transparent as all transactions are carried out in front of everyone. Also, the mutual-saving system has been maintained for some generations and therefore participants believe their turn to receive money will come on a promised date in the future. Hence, members generate trust and ‘think about the future’ through social reconnection.

The stories of Namahoro and Didier illustrated a common healing trajectory across participants. Although three exceptions had no social group to heal their distress, the first author revisited them multiple times to follow-up and observed a shift in their experiences, and found that all of them recovered social interaction with neighbours at some point during the research period. The dominant thoughts on the past were transformed through community involvement; once sufferers became involved in a community they began to think more about a future life that the community would bring. They may not think far into the future but envisage a close, tangible, and promising future which they believe will take place.

## Discussion

This paper has elaborated local concepts and experiences of mental distress and healing among northern Rwandans who are marginalised by the mainstream genocide narratives, and described associations between sociality and temporality in distress and healing. Local experiences of distress included four major concepts: *ibikomere* (wounded feelings), *ihungabana* (mental disturbances), *ihahamuka* (trauma), and*kurwara mu mutwe* (illness of the head, severe mental illness). Participants perceived that one stage of distress developed into another, slightly more serious than the last. Social isolation played a significant role in the development as it activated ‘remembering’ and ‘thinking too much’ about the past, resulting in worsened symptoms. Meanwhile, healing pathways commonly traced a process of leaving the past and moving forward towards a future through community involvement. As participants recovered social reconnection, they began to envisage a hopeful and tangible future with others. In short, the experiences of distress and healing in the research population can be explained by two axes, i.e., sociality (isolation – reconnection) and temporality (past – future), which are associated with each other. It traced a trajectory from past-oriented distress in social isolation to future-oriented healing through social reconnection.

### Local concepts and experiences of distress in people of northern Rwanda

Previous studies have reported *ihahamuka* as a representation of post-genocide trauma and suffering in Rwanda; the concept refers to extreme fear and breathlessness, encompassing physiological and psychological symptoms (Hagengimana & Hinton, 2009; Wilson & Hagengimana, 1998). However, our research population, who were victimised by post-genocide massacres rather than the genocide, expressed their suffering using another concept, *ibikomere*, and emphasised social and emotional aspects of distress. The concept refers to ‘wounded feelings’, mostly social isolation and grief due to family loss, which progresses toward a more severe mental illness. Our findings are supported by previous studies from other conflict-affected settings (Ventevogel et al., 2013; Rasmussen, Keatley & Joscelyne, 2014; Hassan et al., 2016).

Young (1997) provided a view of ‘trauma’ and PTSD diagnosis as a social construction rather than a pre-existing clinical entity. Kleinman and colleagues (1997) also suggest that representation, concept and experience of suffering is shaped, as well as transformed, through interactions between global political economy and local culture and society, where the most vulnerable people, such as the poor and the marginalised, suffer from the influence of the power dynamics. Considering this, the reason why our research illustrated different local concepts from prior literature about Rwanda is possibly explained as follows: under the global movement of supporting genocide survivors, the concept of trauma is so strongly associated with the genocide that others cannot relate themselves to it and needed another concept to express their suffering. As pointed by Doná (2019), the concept of trauma, or *ihahamuka*, has formulated the narrative of Tutsi genocide trauma as a representation of national suffering. However, this politically-dominant narrative might have marginalised and silenced the real voice of genocide survivors as well as of other populations who suffer. Moreover, some researchers argue that *ihahamuka* might be an improvised word, or an existing word with a new meaning attached, after the genocide. For example, Wulsin and Hagengimana (1998) wrote: ‘The culture and its language, Kinyarwanda, still lack words for common depressive and anxiety syndromes. Only since the 1994 civil war has a word emerged for PTSD: *ihahamuka* […].’ Bolton (2001) reported that only participants from the community where foreign aid organisations conducted psycho-education of trauma used the term *ihahamuka* and participants from communities which were not influenced by such interventions did not use this term. Reiterating Bolton (2001), in our research, only participants who had received psychoeducation of trauma used the word *ihahamuka*. Others who had never exposed to such education used *ihungabana* to express similar conditions instead. Combined with prior literature, our findings suggest that psychiatric research and psychoeducational programmes, many of which are originated from foreign countries, take a part of constructing ‘local’ concepts of distress through investigation, theorisation and education. At the same time, they potentially marginalise experiences of a population who are not their explicit target and produce a gap between local understandings of distress.

### Sociality and temporality in local experiences of healing

Being attentive to the marginalised population of Musanze, we found that their experiences of distress and healing are characterised by the temporality–sociality association. This became particularly clear in healing pathways that realign the orientation towards a future through social reconnection. The finding echoed previous studies from Rwanda (Dushimirimana et al., 2014; Leonard et al., 2011; Pells, 2011). Why is the local experience of healing future-oriented, and simultaneously, social?

Anthropological theories suggest that African healing shows multiple-temporalities and timelessness (Igreja, 2012; de Jong, 1987; Last, 2000). As Igreja (2003, 2012) documented, people in Mozambique healed the past through healing the present, as past traumatic experiences were re-enacted as a current problem. Last (2000) also suggested that for people in Sub-Sahara Africa, creating a future has a meaning of regaining what was lost during the war, thus it is healing. Our future-oriented healing can also be interpreted as one form of such timeless healing. Our findings also underline that sociality is necessary for timeless healing to happen. The classic work by Evans-Prichard (1937), and later de Jong (1987), documented the important role played by the audience in healing rituals, drawing from their fieldworks in Azande (North Central Africa) and Guinea-Bissau. The audience helps the patient to get into a trance as well as back to his/her identity. Our research described healing in everyday setting (rather than trance) and reemphasised the important role of others. This highlights that outside formal rituals, settings or interventions, healing equally needs others to be present and involved.

For further understanding, we shed light on the role of ‘social practice’ as pivotal to future-oriented healing. In our research, healing took place in everyday life, through social practices such as church activities and mutual saving. Through the social practices, sufferers reconstructed their life, and continued to live, so that time was opened towards a future. By contrast, isolation triggered ‘remembering’ and ‘thinking too much’ about the past, resulting in a development of mental illnesses. One possible way to theorise this social practice–temporality association is to draw from Schütz’ (1962) phenomenological thinking. In his view, practice is an ‘action’ that is currently taking place and leading the actor towards a future, whilst reflection gives meaning to an ‘act’ that has already been accomplished in the past. Our findings could be interpreted by his theory, and at the same time, offer an additional insight; the practice involving an action happens with, or for, others, whilst reflection (experienced as remembering and thinking too much) happens in isolation. Piaget’s (1971) psychological schema theory also furthers this theorisation. Schema theory explains that existing cognitive schemas are reconstructed when a new experience does not fit the existing schema; as a schema is connected to an action, schema change happens through experiencing new environment. In the light of this theory, leaving the past and moving forward can be interpreted as a form of schema change accommodating a new social experience.

## Conclusion

Our findings showed bottom-up, community-driven healing pathways as an alternative to existing biomedical and psychosocial models. That is, healing the past through creating a future with others. It is through living with others that sufferers can transform the way they experience traumatic past, create a future and heal. 

One important contribution of this study is to urge policymakers, aid-workers and researchers (including ourselves) to be reflexive of their own role in constructing ?local? concepts and experiences of distress through investigation, theorisation and education, and unwittingly marginalising others than the target population. One way of preventing this would be to target a whole geographical area, rather than a selective social group or category, and conduct a mapping of variations of people and their experiences within the area, at an early stage of research and programme planning.

Another contribution is to advance the theory of healing by revealing the association between sociality and temporality. Mental health and psychosocial support programmes in conflict-affected settings mostly take social approaches. Some of them focus on talking about the past and working through the traumatic memory based on evidence of psychological cognitive theory. Others facilitate creative activities that are also reported to be effective on mental health and wellbeing, without knowledge of ?how it works? (Tol et al., 2011). Our findings offer one possible theoretical foundation to explain this; locally-experienced distress emphasises an impact of loss, therefore, a future creation through social practice can heal the past as it retrieves what was lost in war. Hence, we suggest that programmes which facilitate social practice and future creation are also likely to be therapeutic; such programmes could be an option to people who cannot benefit from talking-based, past-oriented or trauma-focused approaches.

## Notes

1 It is pronounced ‘*abachengezi*’.

2 All ethnicities refer to pre-genocide identities, as all citizens are now ‘Rwandan’.
